# Evaluation of *Melia azedarach* extract-loaded poly (vinyl alcohol)/pectin hydrogel for burn wound healing

**DOI:** 10.1371/journal.pone.0270281

**Published:** 2022-06-23

**Authors:** Jun Seung Lee, Kyung Hoon Sun, Yongjin Park

**Affiliations:** Department of Emergency Medicine, School of Medicine, Chosun University, Gwangju, Korea; University of California Davis, UNITED STATES

## Abstract

**Background:**

In this study, a hydrogel comprising poly (vinyl alcohol)/pectin (PVA/PET) was prepared by the addition of *Melia azedarach* extract for epithelial restoration. *M*. *azedarach* extract (MAE) contains volatile organic plant-derived compounds with antimicrobial properties. MAE has a variety of physiological properties, including antimicrobial, insecticidal, and anti-inflammatory activity. This study aimed to investigate whether MAE-loaded PVA/PET hydrogels have protective effects against burn wound healing.

**Methods and findings:**

To mix *M*. *azedarach* with the gel, nanoparticles containing *M*. *azedarach* were prepared using chitosan/maltodextrin as the wall material. A PVA/PET hydrogel containing *M*. *azedarach* was developed and its applicability as a wound dressing was evaluated. In the in vitro scratch assay, MAE treatment showed a scratch recovery-promoting effect comparable to that of the positive control TGF-β1. The MAE-PVA/PET hydrogel was found to be non-toxic, and the antibacterial activity of the hydrogel was excellent against both gram-positive and gram-negative bacteria. Furthermore, as the formulated hydrogel demonstrated strong antimicrobial activity, its wound-healing efficacy was investigated in vivo using a rat model.

**Conclusion:**

MAE was found to be effective against burn wounds and to have antimicrobial activity in vitro and in vivo.

## Introduction

Burns refer to damage to the skin caused by heat, such as sparks, boiling water, chemicals, electricity, or radiation. The lesions are caused by mechanisms such as denaturation of cell proteins due to heat, coagulation, inactivation of enzymes, release of various inflammatory mediators from burnt tissue, and an increased chance of infection due to abnormalities in the immune system. Wet dressings, which have a higher skin regeneration effect than traditional gauze-type dressings, are being used for wound treatment; and the wound dressing market is expected to show a high growth rate in the future. Previously, when treating wounds, it was common to apply a disinfectant until the wound was closed and passively covered with gauze. Traditional dressings depend on the self-renewing ability of the damaged area, however, there is a limit to passive tissue regeneration; therefore, there is an increasing need for a treatment that can effectively regenerate it. Various materials and forms are being studied to design advanced dressings that can deliver effective substances during each regeneration stage. To stimulate wound healing, passive dressing is essential to maintain optimal moisture conditions. Several products, such as gauze, hydrogel, foam, hydrocolloids (carboxymethyl-cellulose), alginate, collagen, cellulose, cotton/rayon, and transparent films (polyurethane), are recommended as passive dressings for wounds and burns because of their influence on the local cellular response [1]. The hydrogel dressing is composed of a water-insoluble cross-linked polymer with high affinity for aqueous media and is a three-dimensional viscoelastic network, which may be composed of a homopolymer or a copolymer. The integration of therapeutics and wound dressings can accelerate and improve wound healing. For this purpose, dressings containing active substances, such as therapeutic agents, are being studied. Antibiotics can be used to prevent infection, and growth factors (platelet-derived growth factors or fibroblast growth factors) can promote tissue regeneration. Vitamin and mineral supplements can help eliminate dead tissue. Hydrocolloids, hydrogels, and bioactive dressings have been investigated for the delivery of these active substances. Research using various materials, systems, and drugs for effective drug release and delivery is ongoing. In addition, dressing products containing antibacterial substances are used to heal infected wounds, and research on materials such as silver, chlorhexidine, honey, methylene blue, gentian violet, and copper has been conducted for antibacterial purposes [2, 3].

Conventional medicinal or cosmetic hydrogels cause skin problems owing to the addition of synthetic preservatives, such as sodium azide and methyl paraben, causing inflammation, sensitive skin, and crushing. In addition, research on natural preservatives is being actively conducted, and components such as golden, green tea, and grapefruit seed extracts, which are known to be natural preservatives, are used; however, they are expensive and have limitations in their application. It encapsulates the components of *Melia azedarach*, which can act as a natural antagonist. When added to a hydrogel, it can be utilized in many procedures because of its low in vivo toxicity and hypoallergenic properties, especially when applied to the skin damaged by the image; and it can also be used as a secondary infection control agent.

Thus, in this study, volatile components in *M*. *azedarach* were formulated in capsule form to improve dispersibility and applicability and were applied to hydrogels. Natural polymer pectin (PET) and synthetic polymer poly(vinyl alcohol) (PVA) were hybridized to produce a hydrogel for use as a dressing material. *M*. *azedarach*-containing hydrogels can be expected to form a physical membrane on the wound site, especially in the medical field, and simultaneously protect the skin from external infections to help repair wounded skin. We also investigated the antimicrobial properties of hydrogels containing *M*. *azedarach* and examined their potential as therapeutic components for wound restoration.

## Methods

### Preparation of MAE

Crude *M*. *azedarach* plant material was purchased from a local store (Gwangju Oriental Herbal Market, Gwangju, Korea) and verified by Professor Sung Dong Cho at the College of Pharmacy, Chosun University. Water-soluble herbal extracts of *M*. *azedarach* were prepared by the Vitabio Corporation, Daejeon, Korea. First, *M*. *azedarach* (100 g) was immersed in 1 L of distilled water and extracted by heating for 2.5 h at 105°C. Then, the extract was filtered using a filter paper (0.45 μm) and stored at 4°C for 24 h. After the extraction process, the fluid concentrate of the herbal extract was centrifuged at 14 240 ×*g* for 15 min and the pH of the collected supernatant was adjusted to 7.0. Finally, the total aqueous extract was filtered using a membrane syringe (0.22 μm), and successive aqueous extracts were stored at –20°C until further use.

### Manufacture of *M*. *azedarach* micro particles and PVA/PET hydrogels

*M*. *azedarach* encapsulation was performed as described by Wanwimol et al. [4] with some modifications. After completely dissolving 1 g of chitosan in 0.1% acetic acid, 10 g of maltodextrin was added to prepare the solution. Twenty grams of *M*. *azedarach* extract (MAE) was added to the chitosan/maltodextrin mixed solution and 1.5 g of Tween 80 was added, followed by emulsification at 7500 rpm for 20 min with a homogenizer (Ika, T25 basic homogenizer). The emulsified solution was dispersed in a 0.1 M NaOH solution (1 mL/100 mL) to solidify chitosan. The solidified particles were centrifuged, filtered, washed with distilled water, freeze-dried for 24–48 h, powdered, and used in the experiment. When adding the hydrogel, the experiment was conducted by adding *M*. *azedarach* capsules at a concentration of 1%. Freezing and thawing were repeated thrice to obtain the PVA/PET hydrogel. The PVA and PET were added to 100 mL distilled water at a ratio of 5:5, mixed with a homogenizer (12500 rpm) for 10 min, and then autoclaved (121°C, 20 min) for dissolution. The dissolved PVA/PET solution was placed in a measuring cylinder to remove air bubbles. An air bubble-free PVA/PET solution was used for the subsequent experiments. The control group comprised the PVA/PET solution without the addition of *M*. *azedarach* capsules, whereas for the experimental group, 15 g of *M*. *azedarach* capsules was added to each petri dish. The petri dishes were allowed to stand for 4 h at –80°C, and then thawed to room temperature for 8 h. This procedure was performed three times to form a hydrogel.

### In vitro scratch assay

For the in vitro scratch assay, HaCaT cells were seeded in 6-well plates at a density of 3 × 10^5^ cells/well and cultured until 80% confluence [5, 6]. The medium was then replaced with serum-free Dulbecco’s modified Eagle’s medium containing mitomycin (10 μg/mL), and the cells were incubated for 2 h to prevent wound proliferation and washed with phosphate-buffered saline. The cell layer was scratched with a sterile 200 μL pipette tip and then rewashed. The wound healing results of the drug treatment group were compared with those of TGF-β1 (100 pg/mL), which was used as a positive control. Increasing the concentration of PGE2 promotes wound healing [7]. Therefore, to evaluate whether *M*. *azedarach* promotes scratch recovery in vitro, HaCaT cells were treated with 2, 10, or 50 μg/mL *M*. *azedarach*, and 48 h later, we evaluated whether the recovery of the damaged area was promoted. One photograph was taken under a microscope (× 100) immediately after scratching, and a second photograph was taken at the same location after 48 h of incubation to observe the wound healing progress. The experiments were repeated thrice, and representative photographs are shown in the figures.

### Antimicrobial evaluation

To measure the antibacterial properties of the PVA/PET hydrogels containing *M*. *azedarach* capsules, the hydrogels were dried under UV light for 5 min, fixed to a thickness of 10 mm, and used for antibacterial experiments. *Staphylococcus aureus* and *Staphylococcus epidermidis* were used as test strains. First, the strain was inoculated in nutrient broth (Difco Co., USA) and cultured at 37°C for 24 h. Each cultured bacteria (1 mL) was mixed in 99 mL of liquid medium, followed by the addition of 10 g sterilized hydrogel. The cells were incubated for 24 h in a shaking incubator. Pure bacterial strains were used as the controls. The experimental groups were divided into a group treated with hydrogel consisting only of the wall material and without *M*. *azedarach*; a group treated with a mixture of hydrogel with *M*. *azedarach* nanoparticles; and a group treated with only hydrogel without *M*. *azedarach* nanoparticles. The turbidity was measured at a wavelength of 700 nm using a UV spectrophotometer to obtain a culture solution for a predetermined time.

### Cytotoxic effect

To measure the cytotoxicity of the hydrogel, a sterile hydrogel based on ISO 10993 was placed in a cell culture solution (Dulbecco’s modified Eagle’s medium, Gibco) and cultured at 37°C for more than 24 h to produce an elution solution. A 96-well tissue culture plate was seeded with RAW 264.7 cells at a density of 1 × 10^5^ cells/mL, treated with hydrogel solution (100 μL), and cultured for 24 h. After 24 h, MTT solution (Sigma-Aldrich Chemical Co., St. Louis, MO) was added to the medium containing the drug, cultured for 4 h at 37°C, and finally, absorbance was measured using an ELISA reader (ELX808, Biotek Instruments, Vermont, USA) at 570 nm.

### In vivo wound experiment

This study conformed to the Guide for the Care and Use of Laboratory Animals published by the US National Institutes of Health (NIH publication number 85–23, revised 1996, latest revision in 2011), and was approved by the Animal Care and Use Ethical Committee of Chosun University (CIACUC2018-S0026). A total of 18 male Sprague Dawley rats (350–400 g, 6–8 weeks old) were housed in individual cages at room temperature (standardized at 26°C) and acclimated for 1 week with standard rat feed. All rats were anesthetized with a mixture of isoflurane, USP (Abbott Labs, Chicago, IL, USA), and oxygen in an induction chamber before the experiment. Once properly anesthetized, the hair on the back of each rat was shaved and the shaved area was cleansed with 70% isopropyl alcohol (BD, Franklin Lakes, NJ, USA). The skin was allowed to dry and equilibrate to the ambient temperature for several minutes. An electric iron was heated to 100°C and placed on the back skin of each animal for 5 s to inflict a superficial burn injury. One hundred microliters of *S*. *epidermidis* suspension (4 × 10^6^ CFU/mL in 1% peptone) were injected into the wound area. The rats were divided into three groups, with six rats per group. The three groups were treated with different hydrogel wound dressings containing sterilized water gauze, hydrogels without *M*. *azedarach* capsules, or hydrogels with *M*. *azedarach* capsules (Fig 4B). Sterilized gauze (Tegaderm; 3M Health Care, USA) was placed on top of the hydrogel and fixed using a flexible band (Coban; 3M Health Care, USA). Rats were injected subcutaneously with 0.02 μg/kg fentanyl citrate (Abbott Labs, Chicago, Illinois, USA) twice daily to reduce pain from burns after burn injury. After 48 h of hydrogel application to the burn wound, the wound site was examined visually and histologically. For histological examination, the animals were euthanized via cervical dislocation under anesthesia, and tissues containing the entire wound were collected and fixed in 10% neutral formalin solution for 24 h. After fixation, tissues were dehydrated and embedded in paraffin cubes. The embedded tissue was cut using a microtome and attached to the surface of an adhesive slide coated with polysine. After paraffin removal, the tissue was stained with hematoxylin and eosin Y to observe the sample under a microscope.

### Statistical analysis

All experimental results are presented as mean ± standard deviation (SD). The differences between the control and experimental groups were compared using Student’s *t*-test. Differences were considered statistically significant when the *p*-value was less than or equal to 0.05.

## Results

### In vitro scratch assay

**Fig 1:**
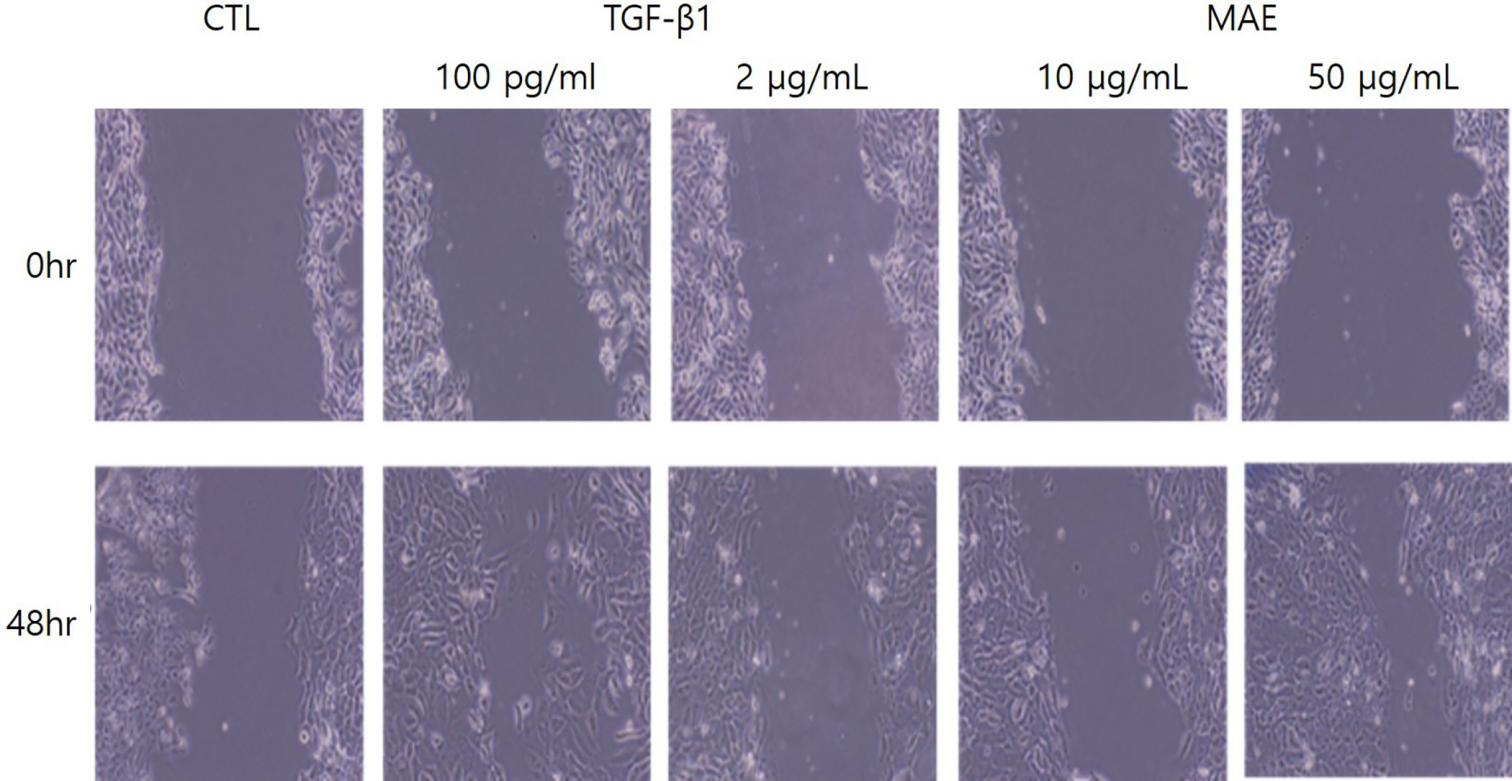
HaCaT cell migration assay. HaCaT cell migration was measured with a wound scratch assay. Treatment with *Melia azedarach* extract (MAE, 50 μg/mL) showed a scratch recovery–promoting effect comparable to that of the positive control TGF-β1 (100 pg/mL). The wound healing efficacy of MAE was confirmed to have an activity equal or superior to that of TGF-β1, which is known to have a conventional wound healing effect.

Treatment with *M*. *azedarach* (50 μg/mL) showed a scratch recovery–promoting effect comparable to that of the positive control TGF-β1 (100 pg/mL) (Fig 1). The wound-healing efficacy of *M*. *azedarach* was confirmed to be equal to or superior to that of TGF-β1, which is known to have a conventional wound-healing effect (Fig 1).

### Antibacterial properties of PVA/PET hydrogels

**Fig 2:**
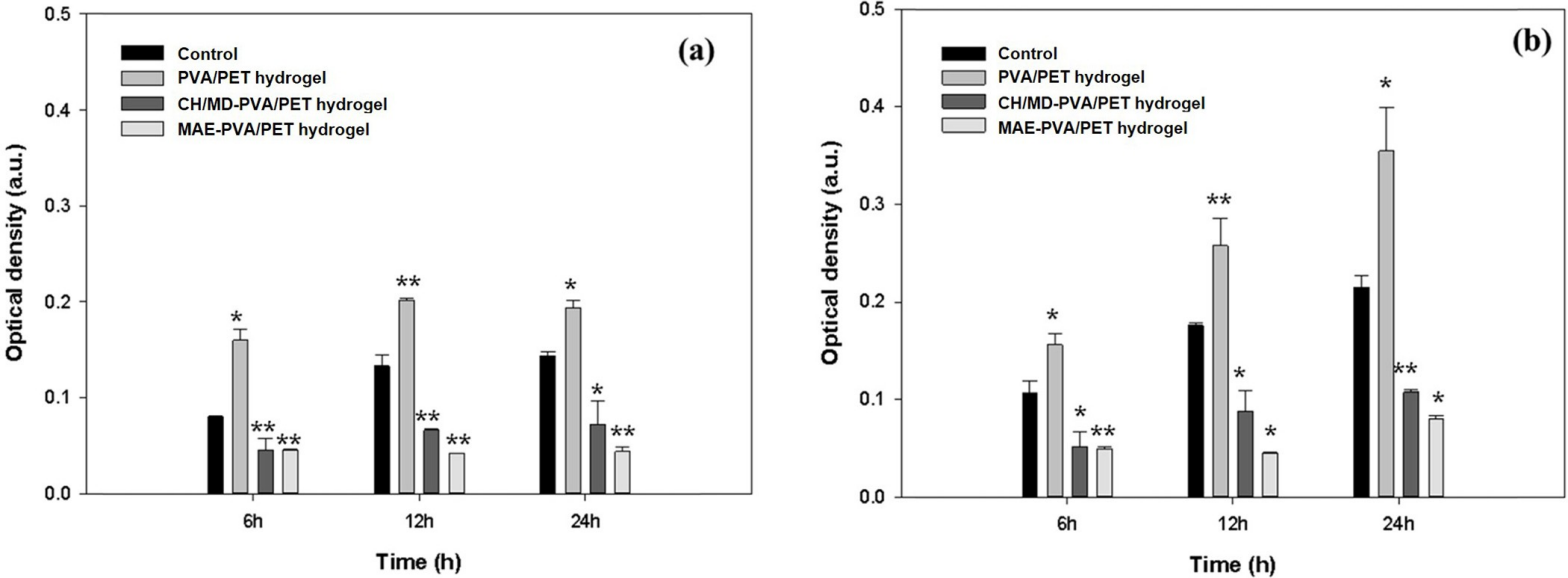
Antimicrobial evaluation. Antimicrobial activity of the *Melia azedarach* extract (MAE)-poly(vinyl alcohol)/pectin (PVA/PET) hydrogel at different exposure times against (a) *Staphylococcus aureus* and (b) *Staphylococcus epidermidis*. *p < 0.05, **p < 0.01 with control and experiment.

To confirm the antibacterial effect of the hydrogel containing the *M*. *azedarach* capsule, each of the prepared gels was added to broth inoculated with *S*. *aureus* or *S*. *epidermidis*, and the bacterial inhibitory ability was evaluated. Fig 2 illustrates the optical density (OD) value of the liquid medium in the control group inoculated with bacteria only, and the groups with the addition of the PVA/PET gel, and the chitosan/maltodextrin material used as a wall material to encapsulate *M*. *azedarach*. This graph compares the inhibition values after incubation with only the PVA/PET gel, and PVA/PET gel with *M*. *azedarach* capsules. Bacterial growth was inhibited in the groups to which MAE capsules were added. Even in the group with added gel, where the wall material was added, the antibacterial effect of chitosan was observed owing to its antibacterial activity. In addition, in the group treated with only PVA/PET gel, the PVA/PET composition became a nutrient that was helpful for bacterial growth and showed a higher OD value than that of the control group, thereby increasing the permeability value.

### Cytotoxicity of PVA/PET hydrogels

**Fig 3:**
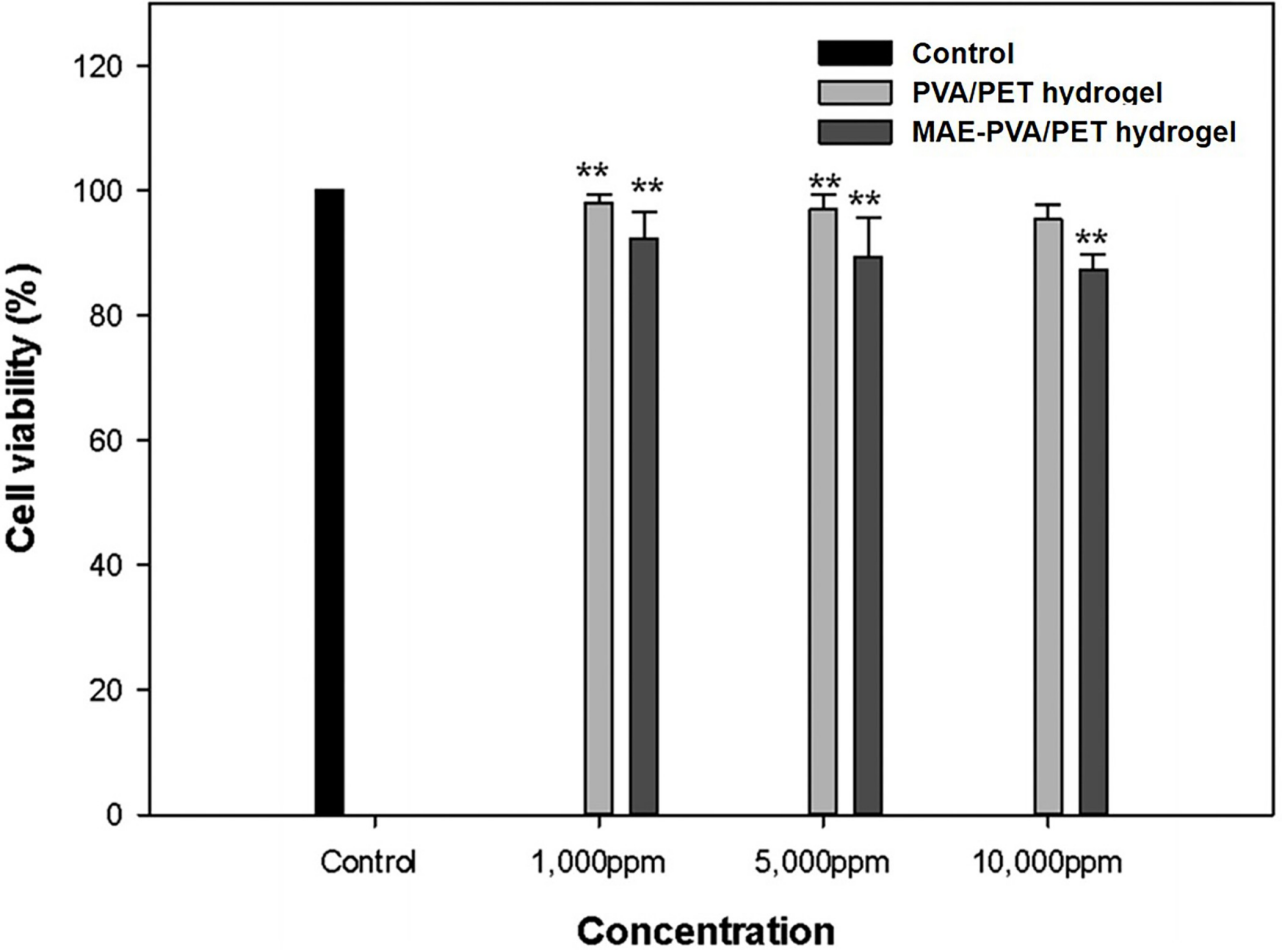
Cell viability assessment. Cell viability of RAW 264.7 cells treated with the extracts of the poly(vinyl alcohol)/pectin (PVA/PET) hydrogel and PVA/PET hydrogel containing *Melia azedarach* extract (MAE) nanoparticles. *p < 0.05 and **p < 0.01 for comparisons between the control and experimental groups.

The cytotoxicity of the eluate was evaluated using an MTT assay for each concentration of *M*. *azedarach* capsule-containing hydrogel. As shown in Fig 3, no toxicity was observed at concentrations ≥ 95%. The cells exhibited a survival rate of > 95% after treatment with each concentration. Therefore, the gel used in this experiment can be used as a non-cytotoxic biomaterial, and its application in various biomaterials should be further investigated.

### Histological evaluation of burn wounds

**Fig 4:**
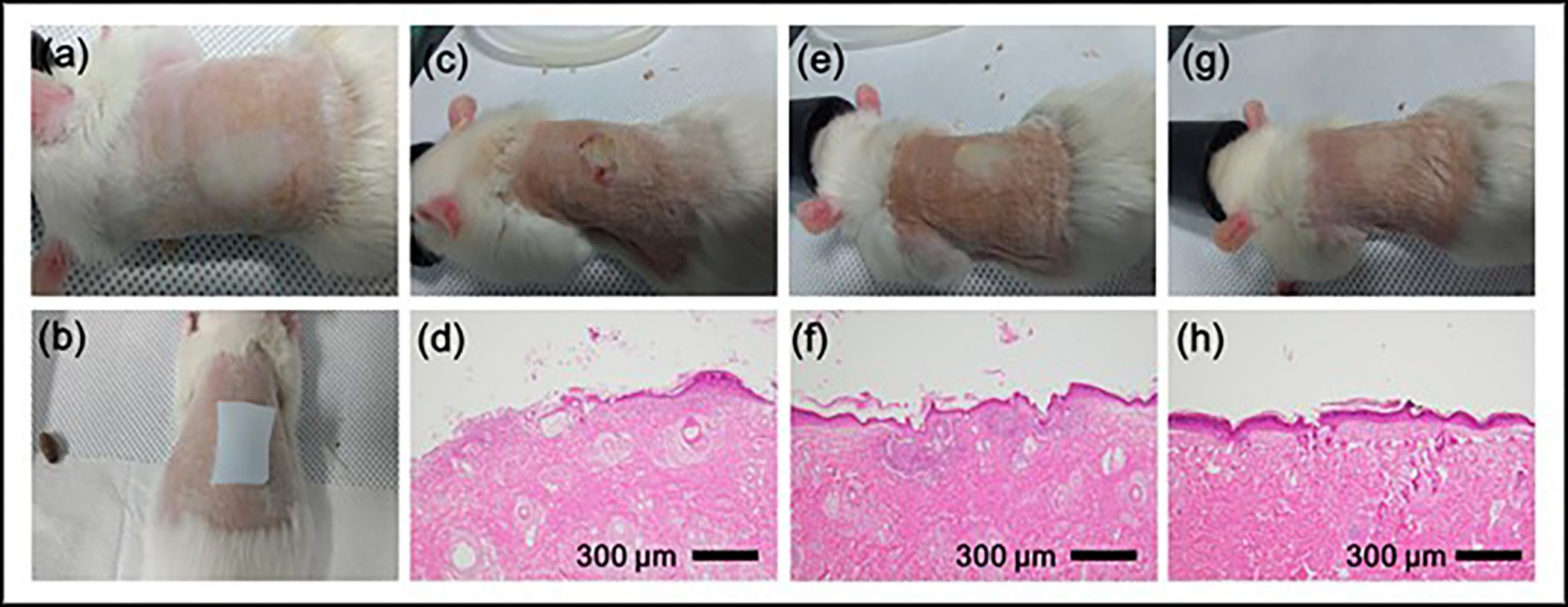
Rat model of wound healing. The back skin of the rats was exposed to a third-degree burn using an iron (dotted circle in (a)). The wound area was covered with three kinds of wound dressings: (b) sterilized water gauze (c and d), hydrogel without *Melia azedarach* extract (MAE) (e and f), and hydrogel with MAE (g and h). After 48 h, the wound surface was observed with the naked eye (c, e, and g), and examined histologically (d, f, and h).

*M*. *azedarach* exhibited stronger antibacterial activity than the hydrogel; however, there was no significant difference in the wound area with the other treatments (Fig 4E and 4G). For a more detailed observation, we performed histological examination of each wound. As shown in Fig 4D, an epithelial layer did not form in the absence of an antimicrobial substance, and traces of pus were observed within the dermis. The wounds treated with hydrogels containing *M*. *azedarach* appeared similar in appearance, but histological examination revealed clear differences. In the case of wounds treated without hydrogels containing *M*. *azedarach*, which has weak antibacterial ability, the epidermidis was not sufficiently formed and was relatively thin, and traces of pus were still observed in the dermis. In contrast, wounds treated with hydrogels containing *M*. *azedarach* capsules fully recovered. Thick epithelial tissues were formed and no traces of pus were found.

## Discussion

The skin, which accounts for approximately 15% of the total body weight, prevents drying in the human body, protects the internal body structure from the external environment, and has a multilayered structure comprising the stratum corneum, dermis, and subcutaneous layer. The stratum corneum is composed of keratinocytes and serves as a major barrier to external environmental factors, such as UV rays, pathogenic microorganisms, and mechanical disturbances. The immediate adjacent layer is the dermal layer, made up of connective tissues comprising collagen and elastin and consists of extracellular matrix, nerve endings, and blood vessels. The deepest layer of the skin is the subcutaneous layer, which is composed of adipose tissues and is responsible for thermal insulation and mechanical protection of the body [8]. Wounds are representative of skin disease and are described as damage and disorders of the skin structure and function, which are the physical barriers of the body. Skin wound healing is a complex process that consists of four steps. The first step is hemostasis, the process by which bleeding is contained at the wound site. The second step, the ’inflammatory phase’, which occurs immediately after injury and involves inflammation, lasts between 24 h and 4–6 days. This phase involves recruitment of proteolytic enzymes and inflammation induced by infiltrating phagocytes (neutrophils and macrophages) at the wound site. It begins with the release of cytokines that cause wound infections in the skin and soft tissues and can be life-threatening. *S*. *aureus*, *Streptococcus pyogenes*, *Escherichia coli*, and *Pseudomonas aeruginosa* are involved. At this stage, all foreign bodies and tissue debris are removed from the wound site by neutrophils and macrophages to prevent infection. The third step is the proliferation stage, in which new granulation tissue is formed by epithelialization and a new extracellular matrix is ​​created at the wound site. At this stage, tissue repair and wound closure begins; the wound area is filled with granulation tissue, and epithelialization occurs at the edge of the wound. Angiogenesis occurs as well as a microvascular network of new blood capillaries to ensure the supply of oxygen and nutrients to the newly formed tissue. The final stage of wound healing is the regeneration stage, in which the normal dermal structure is restored and the tensile strength of the scar tissue is increased [9]. Wound healing is a physiological process involving multiple factors, but its complexity can lead to multiple irregularities. Due to the complexity of the healing mechanisms involved and the wide variety of wound types, the selection of an appropriate wound dressing for wound healing is important. Successful wound management, combined with the characteristics of the dressing material, requires accurate assessment of the patient and the type of wound. To accomodate all aspects of wound healing, wound dressings have various shapes and characteristics, and various types such as films, foams, hydrogels, and hydrocolloids have been developed and used according to the complexity of the wound. An ideal wound dressing should promote rapid healing, with minimal patient discomfort, adherence to damaged tissue, maintenance of balanced phase wetness, oxygen exchange, protection against external infectious agents such as bacteria, and healing of an optimal microenvironment that can accelerate the process. In addition, wound dressings should be flexible and easy to remove [10]. In the traditional approach to treat wounds, wet dressings, which maintain a moist environment, are used. Recently, dressings have been researched and developed to deliver various active substances. Dressing agents for wound treatment are selected according to the types of wounds, and various systematic studies are being conducted owing to the recent development of materials and technologies. Some natural products with emollient, demulcent, epithelializing, astringent, antimicrobial, anti-inflammatory, and antioxidant properties can improve the wound-healing process [11]. Hydrogels (in particular, amorphous hydrogels) consist of more than 80% moisture, with carboxymethylcellulose and calcium alginate as the three-dimensional structural material, which allows these hydrogels to penetrate any space equally; the hydrogel does not flow out of the wound and has the ability to coagulate. This advantage makes hydrogels suitable for the treatment of various dry and necrotizing tissues [12].

Herbal medicines or other traditional medicines have been used as remedies for infectious diseases for thousands of years because of their considerable anti-inflammatory and antimicrobial activities and low degree of side effects. In 2001 and 2002, nearly a quarter of the baseline medicines globally were natural products or derivatives [13]. Recent research in China has shown that these herbs account for 10% of prescription drugs [14]. Several traditional herbs and their derived single ingredients have exhibited a wide range of antimicrobial effects against various pathogens, including antioxidant, anti-inflammatory, antibacterial, antifungal, and immunomodulatory effects [15]. The tree *Melia azedarach* (Family: Meliaceae) is locally recognized as a bakain or drek (Hindi), Persian lilac or China tree (English), and Fleurs lilas (French). In South America, it is generally known as “paraiso” or paradise, and in the US as the Indian lilac or white cedar [16]. The whole plant or its specific parts (leaves, stems, and roots) are known to have medicinal properties and an extended history of use by indigenous and tribal people in India. The extract is used in Ayurvedic medicine in India and Unani medicine in Arab countries for its antioxidant, analgesic, anti-inflammatory, insecticidal, rodenticidal, antidiarrheal, deobstruent, diuretic, antidiabetic, cathartic, emetic, antirheumatic, and antihypertensive activities [17]. Although there are numerous reports on the immunomodulatory properties of *M*. *azedarach*, its therapeutic application as a dressing material in burn wounds has not been studied [18]. *M*. *azedarach* extract has shown important antibacterial activity against many pathogens. These results could be attributed to the presence of previously characterized phenolic compounds and flavonoids [19]. The observed antimicrobial effect may be the result of the basic/synergistic action of anti-inflammatory and cytotoxic components present in the *M*. *azedarach* leaf extract, such as gallic acid.

This study revealed that healing was performed according to the standard steps; at 2 days post-operation, the burn wound was clinically and histologically normal. Wound healing passes through four phases: the initial stage starting at the moment of injury, which is a coagulation process that holds back blood loss; then, a stage of inflammatory reaction and debridement of the wound area, followed by cellular proliferation, tissue remodeling, and collagen deposition, which constitute the repair stage [20]. For complete wound healing, persistent interactions occur at the cellular level, as well as at the cells and extracellular matrix, allowing the healing steps to finish [21]. Various cells, such as leukocytes, monocytes, macrophages, fibroblasts, endothelial cells, and epidermal cells, play a vital role in the healing process to revert the architecture of the skin to its original form. The antibacterial effect of MAE against both gram-positive and gram-negative bacterial strains demonstrates its potential for future application as a broad-spectrum antibiotic in bandages, dressings, or gels. Bacterial biofilms provide a barrier to antibiotic resistance, and microorganisms thrive under this protection. MAE not only penetrates the bacterial cell wall, but also disrupts the biofilm barrier. To determine the prospects for use in wound dressings, the wound healing potential of MAE was assessed using a cell scratch assay. MAE accelerated PGE2 production and promoted wound healing. This indicates a potential aid in wound healing. The application of plant-derived MAE in wound healing and its mechanism of action have also been demonstrated in vivo [22, 23]. In our study, MAE showed negligible toxicity. However, it is necessary to verify its safety and therapeutic effects on the human body.

## Conclusion

Based on the above results, MAE is thought to induce skin stabilization after burn injury by inhibiting the growth of bacteria in the wound and alleviating skin damage.

## Supporting information

S1 Table (XLSX)
